# Supplementary material for: Transcriptome analysis of Kentucky bluegrass subject to drought and ethephon treatment
Source: PLoS One. 2021 Dec 16;16(12):e0261472. doi: 10.1371/journal.pone.0261472 (PMC8675742; doi:10.1371/journal.pone.0261472)
Supplement: Attachment — Submitted filename: Comments for PONE-D-21-16354.docx [file pone.0261472.s008.docx]

**Comments:**

The manuscript needs to address the following major concerns:

**Materials and methods**

**In the sub section 2.1**, authors have discussed about the well-watered control plants with ethephon pre-treatment (ETH). However, authors did not provide any data related to this group.

**In the sub section 2.3**, authors should provide manufacturer’s country for the TRIzol kit used for RNA isolation.

**In the sub section 2.6**, authors should provide manufacturer’s name and country for qPCR kit used for two-step qRT-PCR detection.

**In the sub section 2.6**, authors should mention the citation for evaluating the relative quantitative data using the 2^-ΔΔ CT^ method.

**Results and discussion**

**In the sub-section 3.3**, please properly mention the different sub-figures of Fig. 3 in the text while explaining the figure.

**In the sub-section 3.5**, please correct the sentence by adding ‘regulation of’ in the line “with drought by mediating the regulation of fewer genes in response to drought”.

**In the sub-section 3.5.1**, please mention the plant in which ERF113 have been characterized.

**The manuscript needs to be proofread**. **Please correctly frame the sentences.**

**e.g.** “Type II PI(4,5)P2 phosphatase (Transmembrane protein 55A, 4.7 fold) identified………..transduction pathways”.

“Ras superfamily of small GTP-binding proteins (GTPases) the involvement of ARFs in …………….stresses in plant species”.

“Transgenic *Arabidopsis* plants overexpressing heterologous remorin gene………..including dehydration and salinity”.

“They are among the plant proteases that are increased in their activity following stress”.

“Abiotic stresses drive …………including salt, anoxia, hypoxia, heat, mechanical damages, drought, cold, and waterlogging”.

“During drought…………………..internal turgor pressure is important”.

“In addition, pectin may ………………by modulation of stomata movement”.

**The acronym for ADP-ribosylation** factor-like protein is ARL and please correct the line as “ARF and ADP-ribosylation factor-like protein (ARL) belong to Ras”.

**Please italicize the scientific name of plants in the entire manuscript.** e.g. “*Spartina alterniflora* Loisel” “*Arabidopsis*”, *Oryza sativa*

**Proofread the entire manuscript for typographical and grammatical errors.**

**e.g.** “Tanscript of a remorin gene…..” correct the spelling of Transcript.

“also serves as a precursor for the syntheses of Methionine (Met), glutathione” correct the spelling of synthesis.”

“Plastocyanin like protein (3.1 fold) is evolved in electron transport and…..” correct the spelling of involved.

“Plant defensins are small, highly stable, cysteine-rich peptides constitute a part of the innate…… pathogens”.

“ETH treatment caused up-regulation of genes under drought associated with protein degradation and stabilization.”

“Transport across intracellular membranes………………….mediated by membrane amino acid transporters generally differ in substrate selectivity and affinity”.

“The VGAT is initially……………….. know as the amino acid/auxin permease superfamily”.

*“Sulfite exporter TauE/SafE* gene were also up-regulated”

“While the mechanisms remains elucidated, one possible role….”

“Photosystem II and electron transport were found up regulated by drought in ETH treated plants……..”

“ETH treatment might help plants maintain higher O2 evolution…..”.

In the line, “Proline dehydrogenase (-2.3 and -2.6) catalyzes the”, **Please mention what – 2.3 and – 2.6 are specifying and mention the gene name given in the RNA seq data.**

In the line, “CER3 (c90612_g1, 2.5 fold) and WAX2 (c135869_g1, Inf)” **what does Inf specify?**

**Figures**

**Fig. 1A** is not mentioned anywhere in the text.

Please mention the **supplementary data** where it is discussed in the text.

In Fig. 6, perform the **statistical analysis** to determine the significance for the differences in the RNA seq and qRT-PCR.

**References**

Author should follow authors guidelines of PLoS One for writing references.

Authors should maintain uniformity while writing the references
