# Supplementary material for: Transcriptome analysis of Kentucky bluegrass subject to drought and ethephon treatment
Source: PLoS One. 2021 Dec 16;16(12):e0261472. doi: 10.1371/journal.pone.0261472 (PMC8675742; doi:10.1371/journal.pone.0261472)
Supplement: Attachment — Submitted filename: Response to reviewers.docx [file pone.0261472.s010.docx]

Dear Editor,

Thank you very much for your letter and the comments about the manuscript that we submitted to PLoS One (PONE-D-21-16354). We have learned much from the reviewers’ comments, which are encouraging and constructive and we also have made corresponding revision after reading the comments and advice. Words highlighted in yellow are the changes we have made in the text. Our response of the comments is enclosed at the end of this letter. If you have any question about this manuscript, please don’t hesitate to contact us.

Sincerely yours,

Lixin Xu

Response to Reviewer #1:

Thanks for your comments on my manuscript. We have revised the paper after reading your comments. The main revisions are listed as follows:

1. In the sub section 2.1, authors have discussed about the well-watered control plants with ethephon pre-treatment (ETH). However, authors did not provide any data related to this group.

**Response**: Discussions related to well-watered control plants with ethephon pre-treatment (ETH) was deleted. In this study, we want to investigate the effect of ethephon on drought tolerance of Kentucky bluegrass by comparing and analyzing DEGs between (ETH_D vs CK) and (Drought vs CK). Therefore, we decided to delete the corresponding part related to well-watered control plants with ethephon pre-treatment (ETH).

2. In the sub section 2.3, authors should provide manufacturer’s country for the TRIzol kit used for RNA isolation.

**Response:** The manufacturer’s country for the TRIzol kit used for RNA isolation has been added.

3. In the sub section 2.6, authors should provide manufacturer’s name and country for qPCR kit used for two-step qRT-PCR detection.

**Response:** The manufacturer’s name and country for qPCR kit used for two-step qRT-PCR detection have been added.

4. In the sub section 2.6, authors should mention the citation for evaluating the relative quantitative data using the 2^-ΔΔ CT^ method.

**Response:** The citation for evaluating the relative quantitative data using the 2^-ΔΔ CT^ method has been added.

5. In the sub-section 3.3, please properly mention the different sub-figures of Fig. 3 in the text while explaining the figure.

**Response:** Revised.

6. In the sub-section 3.5, please correct the sentence by adding ‘regulation of’ in the line “with drought by mediating the regulation of fewer genes in response to drought”.

**Response:** Corrected.

7. In the sub-section 3.5.1, please mention the plant in which ERF113 have been characterized.

**Response:** Revised.

8. The manuscript needs to be proofread. Please correctly frame the sentences.

e.g. “Type II PI(4,5)P2 phosphatase (Transmembrane protein 55A, 4.7 fold) identified………..transduction pathways”.

“Ras superfamily of small GTP-binding proteins (GTPases) the involvement of ARFs in …………….stresses in plant species”.

“Transgenic *Arabidopsis* plants overexpressing heterologous remorin gene………..including dehydration and salinity”.

“They are among the plant proteases that are increased in their activity following stress”.

“Abiotic stresses drive …………including salt, anoxia, hypoxia, heat, mechanical damages, drought, cold, and waterlogging”.

“During drought…………………..internal turgor pressure is important”.

“In addition, pectin may ………………by modulation of stomata movement”.

**Response:** We sincerely appreciated the carefulness and suggestions from the reviewer and revised accordingly.

9. The acronym for ADP-ribosylation factor-like protein is ARL and please correct the line as “ARF and ADP-ribosylation factor-like protein (ARL) belong to Ras”.

**Response:** We have corrected it as suggested.

10. Please italicize the scientific name of plants in the entire manuscript. e.g. “*Spartina alterniflora* Loisel” “*Arabidopsis*”, *Oryza sativa*

**Response:**  Revised as suggested.

11. Proofread the entire manuscript for typographical and grammatical errors.

e.g. “Tanscript of a remorin gene…..” correct the spelling of Transcript.

“also serves as a precursor for the syntheses of Methionine (Met), glutathione” correct the spelling of synthesis.”

“Plastocyanin like protein (3.1 fold) is evolved in electron transport and…..” correct the spelling of involved.

“Plant defensins are small, highly stable, cysteine-rich peptides constitute a part of the innate…… pathogens”.

“ETH treatment caused up-regulation of genes under drought associated with protein degradation and stabilization.”

“Transport across intracellular membranes………………….mediated by membrane amino acid transporters generally differ in substrate selectivity and affinity”.

“The VGAT is initially……………….. know as the amino acid/auxin permease superfamily”.

*“Sulfite exporter TauE/SafE* gene were also up-regulated”

“While the mechanisms remains elucidated, one possible role….”

“Photosystem II and electron transport were found up regulated by drought in ETH treated plants……..”

“ETH treatment might help plants maintain higher O2 evolution…..”.

**Response:** We proofread the entire manuscript and revised accordingly. We really appreciated the reviewer for the time spent on this manuscript.

12. In the line, “Proline dehydrogenase (-2.3 and -2.6) catalyzes the”, Please mention what – 2.3 and – 2.6 are specifying and mention the gene name given in the RNA seq data.

**Response:** Revised. Should be “Two proline dehydrogenase unigene (-2.3 and -2.6) catalyzes......”.

13. In the line, “CER3 (c90612_g1, 2.5 fold) and WAX2 (c135869_g1, Inf)” what does Inf specify?

**Response:** If the normalized readcounts of one particular gene in one sample is 0 and not 0 in another sample, fold change would be Inf or -Inf.

14. Fig. 1A is not mentioned anywhere in the text.

**Response:** Fig 1A (Fig 1 in revised manuscript) was mentioned the sub section 2.1 for methods explanation.

15. Please mention the supplementary data where it is discussed in the text.

**Response:** We have mentioned supplementary data in the text where it has been discussed.

16. In Fig. 6, perform the statistical analysis to determine the significance for the differences in the RNA seq and qRT-PCR.

**Response:** Instead of carrying out a statistical analysis by LSD value for the same gene between RNA-Seq results and qRT-PCR expression, we performed a correlation statistical analysis. The correlation between RNA-Seq results and qRT-PCR expression was analyzed to get Pearson’s correlation coefficients as describe in sub section 3.4 (Pearson’s r = 0.98 and P <0.001).

17. Author should follow authors guidelines of PLoS One for writing references.

**Response:** We have revised the references accordingly.

18. Authors should maintain uniformity while writing the references

**Response:** References are revised.

Response to Reviewer #2:

1. Most conclusions are exaggerated and are drawn based on RNAseq data with qRT-PCR validation of randomly selected eight differentially expressed genes.

**Response:** Conclusions from our study are drawn based on a verified RNAseq data set combined with analysis from various published data as discussed in the manuscript.

2. The statistical analysis has not been performed for any of the experiments especially for Relative water content (RWC) and Malondialdehyde (MDA) content. Even though statement significantly increased and decreased is used without performing the statistical test. Based on MDA and RWC content, we cannot conclude that ethephon could improve the drought tolerance of Kentucky bluegrass. Few more assays need to be performed before drawing any of the conclusions.

**Response:** The statistical analysis result of Relative water content (RWC) and Malondialdehyde (MDA) content has been added and noted in the corresponding figure. As suggested, we provided additional images to support our physiological data. For Kentucky bluegrass, RWC and MDA are two key and confirmed indicators for drought tolerance and they are used and investigated in many turfgrass studies for drought tolerance comparison and evaluation. The relevant studied have been mentioned in the corresponding results and discussion.

3. Authors need to show representative images of plants before and after drought stress with ethephon treatment, to understand any physiological changes in control, drought and ethephon treated plants.

**Response:** The representative images of Kentucky bluegrass plants in well-watered conditions, drought-stressed conditions and ethephon-pretreated plants under drought-stressed conditions have been added accordingly.

4. Authors have claimed that changes in the cell wall and cuticular wax along with proline, antioxidant enzymes and unsaturated fatty acids levels due to ethephon treatment has improved the Kentucky bluegrass drought tolerance, based on expression levels. Authors need to further support their findings and claims by performing assays and anatomical studies.

**Response:** We agree that with corresponding assays and anatomical analysis, the conclusions of this study would be more consolidated and thoroughly confirmed. Meanwhile, this study could still provide meaningful gene regulation information to understand the mechanism underlying ethephon-promoted drought tolerance of Kentucky bluegrass. To date, there is few research on this particular aspect. We hope this study could lay a basic and informative foundation for more and more detailed and experimental research in the future.

5. Authors need to improve the quality of the figures provided. In Fig.6, provide the name of genes instead of gene I.D’s, as authors have not mentioned which gene I.D represent which gene. Also, need to discuss two downregulated genes (c117236 and c119413) in fig.6.

**Response:** Genes in Fig 6 (Fig 8 in the revised manuscript) were selected randomly to verify the effectiveness of RNA-Seq data for further expression level analysis. Genes that we discussed in the text were selected based on the comparison of DEGs between different sampling groups. Gene I.Ds and corresponding names were listed in the Supplementary Data 6 and 7, and were highlighted accordingly.

6. The objective of this study was to investigate the effect of ethephon on drought tolerance of Kentucky bluegrass and to understand the underlying mechanism by analyzing and identifying genes involved in ethephon mediated drought tolerance improvement, which authors had not provide any mechanism or tentative figure which assimilates the whole study into hypothesis and randomly selected eight differentially expressed genes does not prove or show any mechanism as such. This study is a more theoretical approach than experimental in revealing the mechanism for how ethephon regulates drought response and improves drought tolerance of Kentucky bluegrass.

**Response:** We appreciated the reviewer’s suggestion. The idea to generalize the tentative mechanism into figure is really helpful in improving our manuscript. As suggested, Fig 9 was added to provide a mechanism hypothesis based on results from the whole study .
