# Supplementary material for: Transcriptome analysis of Kentucky bluegrass subject to drought and ethephon treatment
Source: PLoS One. 2021 Dec 16;16(12):e0261472. doi: 10.1371/journal.pone.0261472 (PMC8675742; doi:10.1371/journal.pone.0261472)
Supplement: Attachment — Submitted filename: Reviewer comments.docx [file pone.0261472.s009.docx]

The manuscript titled “Transcriptome analysis of Kentucky bluegrass subject to drought and ethephon treatment”, describe a technically sound piece of scientific research where Illumina RNAseq analysis has been performed on drought and ethephon treated Kentucky bluegrass and the relative water content and malondialdehyde content were measured, with validation of few differentially expressed genes. The authors made all data underlying the findings in their manuscript fully available. The language in submitted articles is clear, correct, and unambiguous. However, some major concerns need to be addressed

- Most conclusions are exaggerated and are drawn based on RNAseq data with qRT-PCR validation of randomly selected eight differentially expressed genes.
- The statistical analysis has not been performed for any of the experiments especially for Relative water content (RWC) and Malondialdehyde (MDA) content. Even though statement significantly increased and decreased is used without performing the statistical test. Based on MDA and RWC content, we cannot conclude that ethephon could improve the drought tolerance of Kentucky bluegrass. Few more assays need to be performed before drawing any of the conclusions.
- Authors need to show representative images of plants before and after drought stress with ethephon treatment, to understand any physiological changes in control, drought and ethephon treated plants.
- Authors have claimed that changes in the cell wall and cuticular wax along with proline, antioxidant enzymes and unsaturated fatty acids levels due to ethephon treatment has improved the Kentucky bluegrass drought tolerance, based on expression levels. Authors need to further support their findings and claims by performing assays and anatomical studies.
- Authors need to improve the quality of the figures provided. In Fig.6, provide the name of genes instead of gene I.D’s, as authors have not mentioned which gene I.D represent which gene. Also, need to discuss two downregulated genes (c117236 and c119413) in fig.6.
- The objective of this study was to investigate the effect of ethephon on drought tolerance of Kentucky bluegrass and to understand the underlying mechanism by analyzing and identifying genes involved in ethephon mediated drought tolerance improvement, which authors had not provide any mechanism or tentative figure which assimilates the whole study into hypothesis and randomly selected eight differentially expressed genes does not prove or show any mechanism as such. This study is a more theoretical approach than experimental in revealing the mechanism for how ethephon regulates drought response and improves drought tolerance of Kentucky bluegrass.

Overall, authors need to perform few more assays with proper statistical analysis which can support the conclusions drawn by authors and submit high-quality figures.
